# Self-Administered Acupressure for Probable Knee Osteoarthritis in Middle-Aged and Older Adults: A Randomized Clinical Trial

**DOI:** 10.1001/jamanetworkopen.2024.5830

**Published:** 2024-04-19

**Authors:** Wing-Fai Yeung, Shu-Cheng Chen, Denise Shuk Ting Cheung, Carlos King-Ho Wong, Tsz Chung Chong, Yuen Shan Ho, Lorna Kwai Ping Suen, Lai Ming Ho, Lixing Lao

**Affiliations:** 1 School of Nursing, the Hong Kong Polytechnic University, Hong Kong SAR, China; 2 Research Center for Chinese Medicine Innovation, The Hong Kong Polytechnic University, Hong Kong SAR, China; 3 Research Institute for Smart Ageing, The Hong Kong Polytechnic University, Hong Kong SAR, China; 4 School of Nursing, Li Ka Shing Faculty of Medicine, The University of Hong Kong, Hong Kong, China; 5 Laboratory of Data Discovery for Health (D24H), Hong Kong SAR, China; 6 Department of Family Medicine and Primary Care, School of Clinical Medicine, LKS Faculty of Medicine, The University of Hong Kong, Hong Kong SAR, China; 7 Department of Pharmacology and Pharmacy, LKS Faculty of Medicine, The University of Hong Kong, Hong Kong SAR, China; 8 Department of Infectious Disease Epidemiology & Dynamics, Faculty of Epidemiology and Population Health, London School of Hygiene & Tropical Medicine, London, United Kingdom; 9 Sports Med Asia, Hong Kong, Hong Kong SAR, China; 10 School of Nursing, Tung Wah College, Hong Kong, China; 11 School of Public Health, The University of Hong Kong, Hong Kong SAR, China; 12 Virginia University of Integrative Medicine, Vienna, Virginia

## Abstract

**Importance:**

The effects of self-administered acupressure (SAA) on knee osteoarthritis (OA) pain remain unclear.

**Objective:**

To evaluate the effectiveness of SAA taught via a short training course on reducing knee OA pain in middle-aged and older adults.

**Design, Setting, and Participants:**

This randomized clinical trial was conducted among community-dwelling individuals in Hong Kong who were aged 50 years or older with probable knee OA from September 2019 to May 2022.

**Interventions:**

The intervention included 2 training sessions for SAA with a brief knee health education (KHE) session, in which participants practiced acupressure twice daily for 12 weeks. The control group (KHE only) received only education about maintaining knee health on the same schedule and duration.

**Main Outcomes and Measures:**

The primary outcome was the numerical rating scale (NRS) pain score at 12 weeks. Other outcomes included Western Ontario and McMaster University Osteoarthritis Index, Short Form 6 Dimensions (SF-6D), Timed Up and Go, and Fast Gait Speed tests.

**Results:**

A total of 314 participants (mean [SD] age, 62.7 [4.5] years; 246 [78.3%] female; mean [SD] knee pain duration, 7.3 [7.6] years) were randomized into intervention and KHE-only groups (each 157). At week 12, compared with the KHE-only group, the intervention group had a significantly greater reduction in NRS pain score (mean difference [MD], −0.54 points; 95% CI, −0.97 to −0.10 points; *P* = .02) and higher enhancement in SF-6D utility score (MD, 0.03 points; 95% CI, 0.003 to 0.01 points; *P* = .03) but did not have significant differences in other outcome measures. The cost-effectiveness acceptability curve demonstrated a greater than 90% probability that the intervention is cost-effective at a willingness to pay threshold of 1 GDP per capita.

**Conclusions and Relevance:**

In this randomized clinical trial, SAA with a brief KHE program was efficacious and cost-effective in relieving knee pain and improving mobility in middle-aged and older adults with probable knee OA.

**Trial Registration:**

ClinicalTrials.gov Identifier: NCT04191837

## Key Points

**Question:**

Is a self-administered acupressure (SAA) training program effective for relieving pain in patients with probable knee osteoarthritis (OA)?

**Findings:**

In this randomized clinical trial of 314 middle-aged and older adults, the SAA group had a significantly greater improvement in the numerical rating scale pain score than the knee health education group over 12 weeks. A cost-effectiveness acceptability curve demonstrated a greater than 90% probability of SAA being cost-effective at a willingness to pay threshold of 1 GDP per capita.

**Meaning:**

These findings suggest that the implementation of an SAA training program is an efficacious and cost-effective approach to relieve knee pain in middle-aged and older adults with probable knee OA.

## Teaser

This randomized clinical trial evaluates the effectiveness of self-administered acupressure on reducing knee osteoarthritis (OA) pain among middle-aged and older adults.

## Introduction

Knee osteoarthritis (OA) is a debilitating condition that is common among people older than 50 years, and 23% of people aged 40 years or older are affected.^1^ Knee OA is associated with long-term health consequences, such as reduced physical fitness, low quality of life, and increased health care utilization.^2,3,4^ Conventional analgesic medications, such as nonsteroidal anti-inflammatory drugs and acetaminophen, are effective for the treatment of knee OA.^5^ However, the use of these agents is limited due to concerns regarding potential gastrointestinal adverse effects. Nonpharmacological treatments such as patient education, exercise, weight loss, and physical therapy produce substantial benefits,^6^ but the delayed therapeutic effects and requirement of significant changes in behavior discourage individuals from receiving such therapeutic measures. Given the limitations of the conventional treatments, complementary health approaches are commonly sought by patients with knee OA.^7^

Self-administered acupressure (SAA) has been used for different pain conditions,^8,9,10^ and it could be an effective treatment for knee OA pain.^11^ Acupressure stimulates the same acupoints as acupuncture with the use of fingers, hands, or elbows, based on traditional Chinese medicine (TCM) meridian theory. The analgesic mechanism of acupressure may primarily involve the suppression of peripheral inflammation through the regulation of inflammatory signal transduction^12^ and pain signal transduction pathway^13^ modulation of ion channels and inhibition of glial cell activation.^14,15^ Recently, clinical trials have attempted to examine the feasibility and effectiveness of SAA for the treatment of knee OA in postmenopausal women^16^ and older patients.^17,18,19^ These studies were limited by a nonrandomized design,^19^ no fidelity assessment,^16^ and high attrition rate.^16^ A randomized clinical trial (RCT) compared the effects of SAA with sham SAA and usual care in participants with knee OA aged 65 years or older,^18^ in which they found, compared with usual care, that the SAA and sham acupressure groups showed significantly greater improvements in the numerical rating scale (NRS) of pain, Western Ontario and McMaster University Osteoarthritis Index (WOMAC) pain, and function scores. However, the tested acupressure protocol was not empirically used for knee pain reduction in clinical practice^20^ but was borrowed from a previous trial targeting sleep disturbance and fatigue in cancer survivors.^21^

Therefore, the effectiveness of the traditionally used acupoints now being used in clinical practice for knee OA remains uncertain. To bridge this knowledge gap, we conducted this RCT to examine the short-term (4-week) and medium-term (8- and 12-week) effectiveness of SAA on traditionally used acupoints, along with health economic analyses, for relieving knee OA pain in middle-aged and older adults.

## Methods

### Setting and Design

This study was an assessor-blinded, 2-group RCT. All eligible participants were randomized to the SAA intervention group or the knee health education (KHE)–only comparison group at a 1:1 ratio. The methods and statistical analysis plan are described in Supplement 1. This study was reported following the Consolidated Standards of Reporting Trials (CONSORT) reporting guideline.^22^ The study protocol was approved by the Hong Kong Polytechnic University Human Subjects Ethics Subcommittee.

### Participants

The participants were recruited from September 2019 to May 2022 through posters in the Hong Kong Polytechnic University, community centers, and social media. Written informed consent was obtained from all participants. Eligible participants aged 50 years or older were able to comprehend Chinese and fulfilled any 3 of the following criteria: (1) morning stiffness for 30 minutes or less; (2) crepitus on active joint motion; (3) bone tenderness; (4) bone enlargement; or (5) no palpable joint warmth. These criteria yielded 84% sensitivity and 89% specificity for OA knee diagnosis in those aged 50 years or older.^23^ Participants also had knee pain for at least 3 months^24^ and rated their pain levels as 3 or greater on the NRS. The exclusion criteria were (1) knee pain related to other conditions (eg, cancer, fracture, rheumatoid arthritis, and rheumatism) according to the red flags for further investigation or referral in the National Institute for Health and Care Excellence Guidelines for Osteoarthritis of the knee^25^; (2) history of acupressure, acupuncture, or steroid injection for knee pain in the past 6 months; (3) previous knee replacement surgery; (4) medical diagnoses or conditions that precluded individuals from active participation (eg, bleeding disorders, alcohol or drug abuse); (5) pregnancy; (6) a body mass index greater than 30 (body mass index is calculated as weight in kilograms divided by height in meters squared)^26^; (7) a score of less than 22 in Hong Kong Montreal Cognitive Assessment^27^; and (8) presence of skin lesions or infections at the treatment sites.

### Intervention

The 2-hour training sessions for both groups were held over 2 consecutive weeks, 1 week apart (total of 4 hours; eFigure in Supplement 2). The SAA intervention protocol was developed based on TCM meridian theory by previous studies^16,20^ and modified by the experienced acupuncturists (W.-F. Y. and L.L.). The KHE protocol was developed based on the materials from the websites of Elderly Health Service, Department of Health, Hong Kong SAR^28^ and modified by a physiotherapist (T.C.C.).^17,29^ A brief version of KHE was included in the SAA course to provide a comprehensive coverage. The details of interventions are described in eTable 1 and 2 in Supplement 2. The SAA instructors were registered Chinese medicine practitioners with at least 5 years of clinical experience and trained by the experienced acupuncturists (W.-F.Y. and L.L.), while the KHE instructors were registered nurses and received training from a physiotherapist. The principal investigator (W.-F.Y.) randomly visited the classes to ensure the intervention delivery was in accordance with the protocol. The training was conducted in small groups of 4 to 7 participants. The participants’ acupressure performance, including the rhythm, force, technique, and acupoint location, were inspected by the instructor with a competency checklist in each session. Follow-up phone calls were made twice per week to remind the participants to practice and to answer any relevant queries during the first week.

### Randomization and Masking

Randomization was conducted by an independent researcher by using block randomization with random block sizes of 4 to 6. The treatment allocation was enclosed in sealed, opaque, sequentially numbered envelopes. The envelopes were disclosed after the participants had completed all baseline assessments. Assessors were masked for group allocation.

### Outcomes

Assessments were conducted at baseline and at weeks 4, 8, and 12. The primary outcome measure was pain severity NRS,^30^ which was an 11-point scale ranging from 0 (no pain) to 10 (greatest pain imaginable). Secondary outcomes included WOMAC, used to assess pain, stiffness and physical functional disability^31,32^; and the Short Form 6 Dimension (SF-6D), used to assess quality of life.^33,34^ Timed Up and Go (TUG) and Fast Gait Speed (FGS) tests were used to assess mobility, walking ability, balance, and fall risk.^35,36^

Intervention compliance throughout the 12-week period was assessed by providing a logbook to participants to record their daily acupressure practice (duration and frequency) in the SAA group or compliance to knee health instructions (whether they had followed the 6 instructions) in the KHE-only group. The acceptability of training content was assessed through a 10-point scale item and open-ended questions. Participants were also asked to report any adverse events (AEs) during their implementation of daily practice.

### Cost-Effectiveness Analysis

Quality-adjusted life-years (QALYs) were determined from the SF-6D utility score using the standard area-under-the-curve approach.^34^ Costs of implementation were evaluated from the perspective of the health care system. Hong Kong’s health care system, a dual-track model, comprises a heavily government-subsidized public sector, providing 90% of inpatient services, and a private sector operating on a fee-for-service basis.^37,38^ The incremental cost-effectiveness ratio (ICER) was calculated as follows: different in costs divided by difference in effects, where difference in effects represents the difference in the QALYs (changes in SF-6D utility scores) between the SAA and KHE-only groups. To test the robustness and uncertainty of the ICER, we performed bootstrapping involving 5000 iterations, and the results were plotted in a cost-effectiveness plane. A cost-effectiveness acceptability (CEA) curve displaying the probability that the intervention was cost-effective for a range of willingness-to-pay threshold values was also plotted.

### Statistical Analysis

The primary analysis was using intention-to-treat approach. The changes in the primary outcome (NRS score) and other secondary outcomes were compared using a linear mixed-effects model (LMM) with group-by–time point interaction (baseline to week 12). Missing data were handled by the LMM. Participants’ sociodemographic and clinical characteristics at baseline were examined for potential group differences by *t* test or χ^2^ test. Acceptability and compliance were presented using descriptive statistics. Clinical significance was examined by the proportion of participants who had a reduction of at least 2 points from baseline in the NRS using χ^2^ test.^39^ Significance level was set to .05 for all statistical analyses, and all tests were 2-tailed. Data analysis was conducted with SPSS version 28.0.1.0 (IBM Corp).

## Results

**Figure 1.**
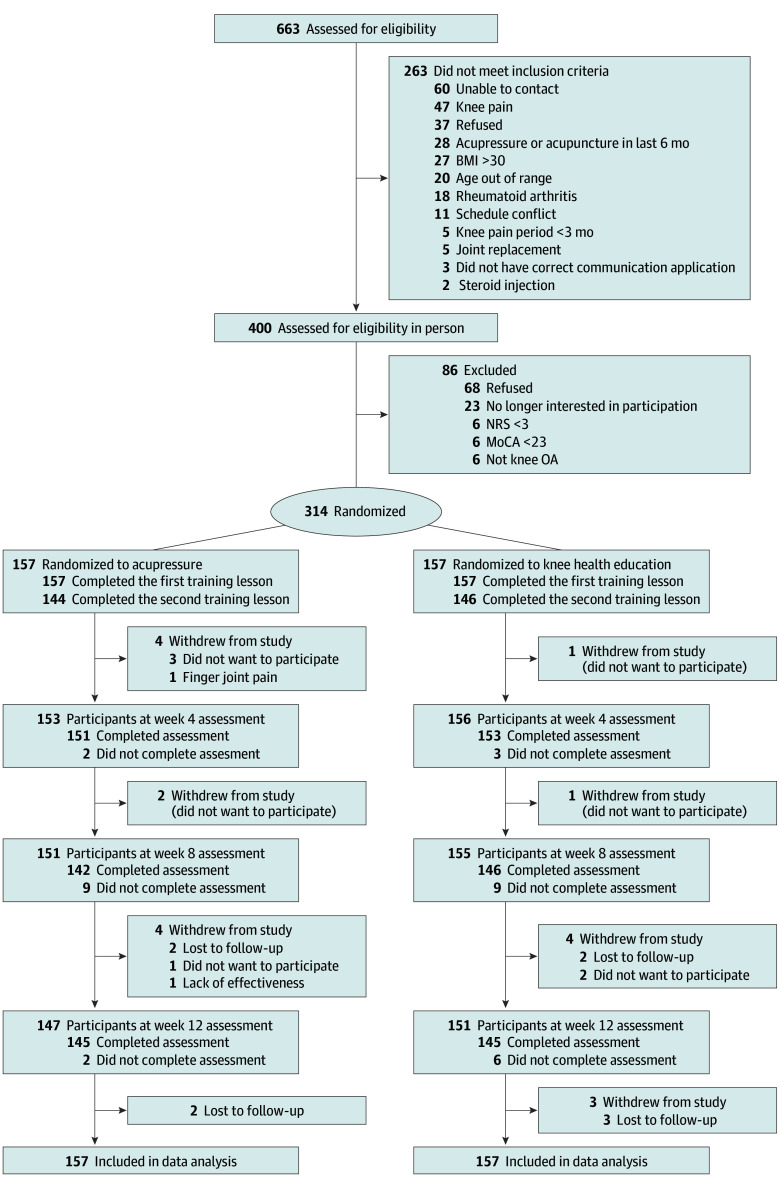
Recruitment Flowchart BMI indicates body mass index (calculated as weight in kilograms divided by height in meters squared); MoCA, Montreal Cognitive Assessment; NRS, numeric rating scale; OA, osteoarthritis.

A total of 314 participants (246 female [78.3%]; mean [SD] age, 62.7 [4.5] years; mean [SD] knee pain duration, 7.3 [7.6] years) were randomized into either the SAA or KHE-only group (157 each) (Table 1). Overall, 12 participants in the SAA group (7.6%) and 9 participants in the KHE-only group (5.7%) withdrew, without significant difference (*P* = .50). The study flow diagram is shown in Figure 1.

**Table 1.** Baseline Characteristics of the Participants

Characteristic	Participants, No. (%)		
	All participants (N = 314)	Self-administered acupressure (n = 157)	Knee health education (n = 157)
Age, mean (SD), y	62.7 (4.54)	62.6 (4.72)	62.8 (4.36)
Sex			
Male	68 (21.7)	34 (21.7)	34 (21.7)
Female	246 (78.3)	123 (78.3)	123 (78.3)
Education level			
≤Primary	28 (8.9)	14 (8.9)	14 (8.9)
Junior high school	60 (19.1)	32 (20.4)	28 (17.8)
Senior high school	122 (38.9)	63 (40.1)	59 (37.6)
≥Bachelor degree	104 (33.1)	48 (30.6)	56 (35.7)
Marital status^a^			
Single	40 (12.8)	19 (12.2)	21 (13.5)
Cohabitation	14 (4.5)	8 (5.1)	6 (3.8)
Married	222 (71.2)	113 (72.4)	109 (69.9)
Divorced	19 (6.1)	8 (5.1)	11 (7.1)
Widowed	17 (5.4)	8 (5.1)	9 (5.8)
Employment status			
Professional or semiprofessional	31 (9.9)	18 (11.5)	13 (8.3)
Skilled worker	17 (5.4)	9 (5.7)	8 (5.1)
Nonskilled worker	7 (2.2)	2 (1.3)	5 (3.2)
Retired	169 (53.8)	82 (52.2)	87 (55.4)
Housewife	69 (22)	35 (22.3)	34 (21.7)
Unemployed	8 (2.5)	2 (1.3)	6 (3.8)
Others	13 (4.1)	9 (5.7)	4 (2.5)
Body mass index, mean (SD)^b^	23.5 (2.87)	23.4 (2.91)	23.5 (2.84)
Presence of health problem^c^	220 (70.1)	120 (76.4)	100 (63.7)
Knee pain duration, mean (SD), y	7.3 (7.58)	7.5 (7.02)	7.1 (8.13)
Current use of pain killers for knee pain	43 (13.7)	20 (12.7)	23 (14.6)
Treatment received for knee osteoarthritis management			
Western medicine	140 (44.6)	76 (48.4)	64 (40.8)
Physiotherapy	119 (37.9)	56 (35.7)	63 (40.1)
Chinese medicine (internal use)^d^	42 (13.4)	22 (14)	20 (12.7)
Chinese medicine (external use)^e^	55 (17.5)	25 (15.9)	30 (19.1)
Supplements^f^	218 (69.4)	109 (69.4)	109 (69.4)

^a^ Two participants were missing data for marital status.

^b^ Body mass index was calculated as weight in kilograms divided by height in meters squared.

^c^ Health problem refers to any health problems other than osteoarthritis.

^d^ Chinese medicine (internal use) refers to prescribed or over-the-counter Chinese herbal medicine products intended for ingestion.

^e^ Chinese medicine (external use) refers to Chinese herbal medicine products intended for external application, as well as acupuncture, moxibustion, and cupping treatments.

^f^ Supplements refer to dietary supplements, including vitamins, minerals, and glucosamine.

### Intervention Compliance and Acceptability

A total of 144 participants (91.7%) and 146 participants (93.0%) completed the 2 training sessions in the SAA group and KHE-only group, respectively. The mean (SD) ratings on degree of willingness to attend similar training courses in the SAA and KHE-only groups were 9.5 (0.9) and 9.3 (1.1), respectively. Of the 146 participants in the SAA group who had returned their acupressure log, 116 (79.5%) performed the acupressure at least 4 days per week during the 12-week period. The duration of self-practice at home was 16.5 minutes per day on average.

### Effectiveness

#### Primary Outcome

The differences in the NRS score between the SAA and KHE-only groups across study time points were compared using the LMM (Table 2; eTable 3 in Supplement 2).^40^ Compared with the KHE-only group, the SAA group had a significantly greater reduction in NRS scores across all the assessment time points, with a between-group difference in changes from baseline to week 4 of −0.59 points (95% CI, −0.96 to −0.21 points; *d* = 0.35; *P* = .002); to week 8, −0.67 points (95% CI, −1.09 to −0.25 points; *d* = 0.35; *P* = .002), and to week 12, −0.54 points (95% CI, −0.97 to −0.10 points; *d* = 0.27; *P* = .02) (Table 2).

**Table 2.** Study Outcomes Across Study Time Points

Time	Mean (SE)^a^		Difference between groups in change from baseline (95% CI)	Effect size, *d*^b^	*P* value^c^
	SAA group (n = 157)	KHE-only group (n = 157)			
**NRS pain**					
Baseline	5.14 (0.15)	5.15 (0.15)	NA	NA	.006^d^
Week 4	3.74 (0.16)	4.33 (0.15)	−0.59 (−0.96 to −0.21)	0.35	.002
Week 8	3.43 (0.16)	4.11 (0.16)	−0.67 (−1.09 to −0.25)	0.35	.002
Week 12	3.04 (0.16)	3.58 (0.16)	−0.54 (−0.97 to −0.10)	0.27	.02
**WOMAC-pain**					
Baseline	6.82 (0.27)	7.59 (0.27)	NA	NA	.60^d^
Week 4	5.66 (0.27)	6.64 (0.27)	−0.22 (0.81 to 0.37)	0.08	.46
Week 8	5.07 (0.28)	6.24 (0.27)	−0.41 (1.09 to 0.28)	0.13	.25
Week 12	5.03 (0.28)	5.86 (0.27)	−0.06 (0.78 to 0.65)	0.02	.86
**WOMAC-stiffness**					
Baseline	2.50 (0.13)	2.99 (0.13)	NA	NA	.33^d^
Week 4	2.02 (0.13)	2.65 (0.13)	−0.14 (−0.46 to 0.18)	0.10	.40
Week 8	1.89 (0.13)	2.30 (0.13)	0.07 (−0.28 to 0.43)	0.05	.68
Week 12	1.91 (0.13)	2.22 (0.13)	0.19 (−0.17 to 0.54)	0.12	.30
**WOMAC–physical function**					
Baseline	21.92 (0.94)	25.38 (0.94)	NA	NA	.77^d^
Week 4	18.55 (0.96)	21.50 (0.95)	0.50 (−1.33 to 2.32)	0.06	.59
Week 8	16.30 (0.97)	20.07 (0.96)	−0.32 (−2.49 to 1.85)	0.03	.77
Week 12	16.17 (0.97)	19.25 (0.96)	0.37 (−1.92 to 2.66)	0.04	.75
**TUG, s**					
Baseline	10.78 (0.15)	10.99 (0.15)	NA	NA	.08^d^
Week 4	9.78 (0.16)	10.36 (0.16)	−0.37 (−0.74 to 0.01)	0.22	.05
Week 8	9.34 (0.17)	10.02 (0.17)	−0.46 (−0.88 to −0.05)	0.24	.03
Week 12	9.48 (0.17)	9.76 (0.16)	−0.07 (−0.48 to 0.34)	0.04	.74
**Gait speed, s**					
Baseline	3.83 (0.07)	3.81 (0.07)	NA	NA	.66^d^
Week 4	3.90 (0.07)	3.80 (0.07)	0.07 (−0.14 to 0.28)	0.08	.49
Week 8	3.85 (0.08)	3.84 (0.08)	−0.01 (−0.23 to 0.21)	0.01	.93
Week 12	3.68 (0.08)	3.72 (0.08)	−0.07 (−0.28 to 0.14)	0.07	.52
**SF-6D**					
Baseline	0.69 (0.01)	0.69 (0.01)	NA	NA	.18^d^
Week 4	0.71 (0.01)	0.70 (0.01)	0.01 (−0.01 to 0.03)	0.08	.46
Week 8	0.72 (0.01)	0.70 (0.01)	0.02 (−0.01 to 0.04)	0.16	.16
Week 12	0.74 (0.01)	0.71 (0.01)	0.03 (0.003 to 0.01)	0.25	.03

Abbreviations: NA, not applicable; NRS, numerical rating scale; SF-6D, Short Form 6 Dimension; TUG, Timed Up and Go Test; WOMAC, Western Ontario and McMaster University Osteoarthritis Index.

^a^ Estimated mean and standard error (SE) from linear mixed-effects model.

^b^ Effect size based on the mean change from baseline in the treatment group minus the mean change from baseline in the control group, divided by the pooled pretest standard deviation. Effect size was considered as small (0.2), medium (0.5), or large (0.8).^40^ A positive effect size denotes a better effect in the intervention group.

^c^ *P* value for interaction between groups and assessment time points in the linear mixed-effects model.

^d^ *P* value for interaction between groups and all assessment time points in the linear mixed-effects model.

#### Secondary Outcomes

WOMAC (pain, stiffness, and physical function) subscores decreased across the time points but without significant between-group difference. Compared with the KHE-only group, a significant better performance in TUG test was observed in the SAA group at week 8, with a between-group difference in changes from baseline of −0.46 seconds (95% CI, −0.88 to −0.05 seconds; *d* = 0.25; *P* = .03) but the differences were marginally insignificant at week 4 and not significant at week 12. The FGS test showed no significant changes across the time points. The SF-6D utility score improved in both groups across time points, and the SAA group showed a significantly higher enhancement than the KHE-only group at week 12 (mean difference, 0.03 points; 95% CI, 0.003 to 0.01 points; *d* = 0.24; *P* = .03).

#### Clinical Significance

A significantly higher proportion of participants in the SAA group showed a reduction of at least 2 points in NRS across study time points. At week 4, 70 SAA participants (44.6%) and 45 KHE-only participants (28.7%) had a clinically significant change in NRS (*P* = .003); at week 8, 79 SAA participants (50.3%) and 57 KHE-only participants (36.3%) (*P* = .01); and at week 12, 97 SAA participants (61.8%) and 77 KHE-only participants (49.0%) (*P* = .02).

### AEs

Overall, 21 participants (13.4%) in the SAA group reported having AEs, including pain at the acupoints (5 participants), finger joint pain (5 participants), bruise at the acupoints (3 participants), worsening of knee pain (2 participants), worsening of crepitus (2 participants), worsening of stiffness (2 participants), thigh muscle pain (1 participant), and calf cramping (1 participants). All the reported AEs were mild and self-resolved, except for 1 participant who dropped out due to finger joint pain.

### Cost-Effectiveness Analysis

The mean (SD) QALYs as measured by SF-6D utility scores from baseline to week 12 were 0.1651 (0.0212) in the SAA group, which was 0.0042 greater than that in the KHE-only group (mean [SD], 0.1609 [0.0235]), but the difference was not significant (*P* = .10). The mean (SD) cost of the SAA intervention per person in the SAA group was HK$698 (HK$152 [mean, US$89; SD, US$19]), which was significantly greater than that in the KHE-only group (mean [SD], HK$652 [HK$105] [mean, US$83; SD, US$13]; *P* = .002). The ICER for the SAA group relative to the KHE-only group was HK$10 874.

**Figure 2.**
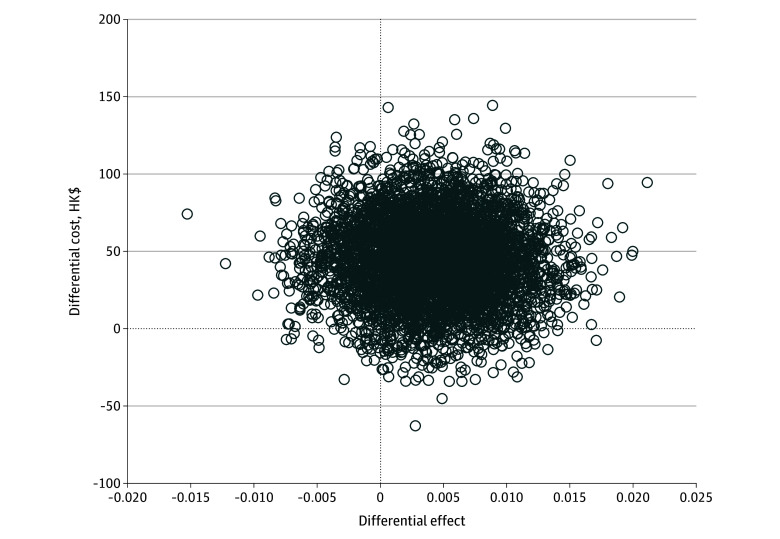
Cost-Effectiveness Plane for Quality-Adjusted Life-Years After Self-Administered Acupressure or Knee Health Education for Knee Osteoarthritis To convert Hong Kong dollars to US dollars, multiply by 0.13.

**Figure 3.**
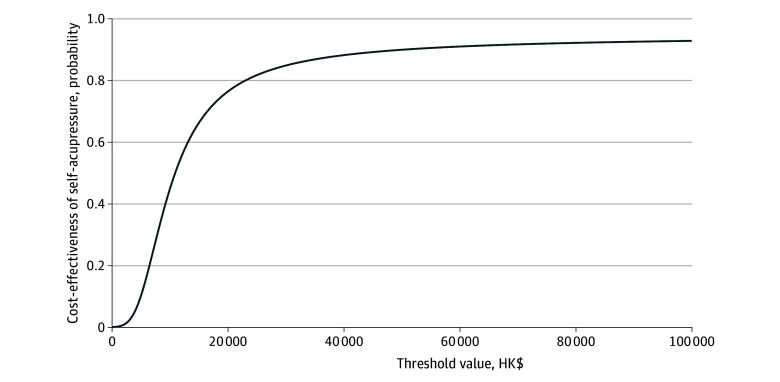
Cost-Effectiveness Acceptability Curve of Self-Administered Acupressure for Knee Osteoarthritis by Varying the Willingness-to-Pay Threshold Values To convert Hong Kong dollars to US dollars, multiply by 0.13.

Sensitivity analysis on the ICER comparing SAA and KHE-only was conducted using 5000 bootstrapping iterations and plotted on the cost-effectiveness plane (Figure 2). Most boostrapped samples were located in the upper right quadrant, suggesting that SAA was associated with a larger improvement in QALY and higher costs than KHE alone. As illustrated by the parametric CEA curve, the probability of SAA being cost-effective reached 80% when the willingness-to-pay threshold was HK$23 000 (US$2948) (Figure 3). Figure 3 demonstrates an 80% probability of SAA being cost-effective at a willingness-to-pay threshold of HK$44 000 (US$5641), and greater than 90% of SAA being cost-effective at willingness-to-pay threshold of 1 GDP per capita (Figure 3).

## Discussion

The results of this large-scale RCT showed that the SAA training program embedded with a brief KHE program outperformed the full KHE program alone in relieving general knee pain in middle- and older-aged adults throughout the 12 weeks. In addition, participants in the SAA group performed significantly better in the TUG test and had a better quality of life in the medium term than those in the KHE-only group. Notably, participants had high acceptability and compliance with the SAA training program. However, the examined SAA training program did not show significantly higher QALYs than the KHE-only group. Even so, our cost-effectiveness analysis supported the SAA program as a cost-effective intervention.

This study, to our knowledge, was the largest RCT that examined the effectiveness of an SAA training program for probable knee OA along with health economic analyses. Our study found a small effect size for the SAA training program in relieving knee pain by NRS compared with KHE alone. Despite decreasing WOMAC scores in both groups, no significant between-group difference was found. These findings aligned with the RCT by Li et al,^18^ which reported significantly greater improvements in NRS scores in both SAA (*d* = 0.42) and sham SAA (*d* = 0.29) groups compared with usual care. However, their report of significantly greater reduction in WOMAC pain score (*d* = 0.53) and WOMAC function (*d* = 0.64) contradicted our results. This discrepancy may be attributed to our use of KHE alone as a comparison group. In our study, KHE-only participants also experienced a decrease in NRS and WOMAC scores over time. Consequently, the between-group differences were smaller than those in the study by Li et al,^18^ which used usual care for comparison.

The KHE-only group also showed an improvement in knee pain and other outcome measures, which narrowed the between-group differences. A previous systematic review on the effectiveness of education programs for knee OA reported inconsistent results,^41^ whereas a recent meta-analysis of group-based education for KOA demonstrated additional benefits only when delivered in combination with exercise.^42^ Therefore, more research is still needed to confirm the findings. Another issue is that the content, development, and delivery of the education programs in previous trials were usually unclear and not thorough,^43^ which may hinder other researchers from replicating the protocol or translation into clinical practice. Future reporting guidelines should be developed for reporting educational interventions.

Given that acupressure training involves much contact time between the instructor and participants, the improvement in participants’ pain, if any, might be attributed to nonspecific effects from the participant-practitioner interaction. Care as usual and waiting list control used in previous studies were not able to control for such nonspecific effects and even led to nocebo effects due to not being treated.^44^ Sham control design has been proposed in SAA studies.^45^ However, the use of sham control in acupuncture-related trials remains controversial, as the intervention characteristics, such as palpation of acupoints and general physical pressure stimulation, may also contribute to the nonspecific effects of the treatment, which can be considered part of the therapeutic elements.^46,47^ These characteristics may not be as clearly discerned as nonspecific effects. Therefore, the present study adopted KHE for the same length as the SAA intervention to control the contact hours between the instructor and participants.

The safety data showed that, consistent with previous findings, SAA is a reliable, well-tolerated nonpharmacological intervention.^48,49^ The most frequently reported AE was finger joint pain, likely due to prolonged or improper pressing the acupoints, which was also found in other studies of SAA.^16,17,50^ Other probable intervention-related AEs are mild and self-resolving but can be prevented by proper acupressure training. To enhance the safety of SAA, future studies should promote the use acupressure rods to prevent finger overuse and overexerting pressure on acupoints.

### Limitations

The present RCT had several limitations. First, the absence of a sham control group and longer-term follow-up limited the ability to discern the specific effects and long-term effects of SAA. However, we adopted a pragmatic approach using KHE alone as a comparison, which allows us to compare the effects of the SAA training program with a clinically used intervention. Second, the absence of objective measures to evaluate the swelling or range of motion of the knee might weaken the validity of the study outcomes, but we had adopted TUG and FGS tests to assess functional mobility. Third, we cannot rule out the potential for social desirability bias, which could result in participants overreporting the benefits. However, given that some outcome measures did not demonstrate significant improvements, the impact of such bias, if present, is likely to be minimal. Furthermore, individuals in the KHE-only group may access external information regarding acupressure and conduct the intervention by themselves, thereby potentially underestimating the treatment effect size of the SAA group.

## Conclusions

In this RCT, we found that a short SAA training program, accompanied with a brief KHE session, could effectively alleviate knee pain and improve mobility in middle- and older-aged adults with probable knee OA. It was noteworthy that participants showed high acceptability and compliance with the SAA training program. Our cost-effectiveness analysis indicated that the SAA was a cost-effective intervention.

## Notes

Supplement 1.
Trial Protocol

Supplement 2. **eFigure.** Schedule of Self-Administered Acupressure Training Course and Knee Health Education Course **eTable 1.** Acupoints Used in the Treatment Protocol **eTable 2.** Self-Administered Acupressure Protocol **eTable 3.** Study Outcomes Across Study Time Points

Supplement 3.
Data Sharing Statement
