# Supplementary material for: Self-Administered Acupressure for Probable Knee Osteoarthritis in Middle-Aged and Older Adults: A Randomized Clinical Trial
Source: JAMA Netw Open. 2024 Apr 19;7(4):e245830. doi: 10.1001/jamanetworkopen.2024.5830 (PMC11031685; doi:10.1001/jamanetworkopen.2024.5830)
Supplement: Supplement 1. — Trial Protocol [file jamanetwopen-e245830-s001.pdf]

**Supplement 1. Study Protocol**

# Title: Self-administered Acupressure for Knee Osteoarthritis in Middle- and Older-Aged Adults: A Randomized Controlled Trial


## Abstract

**Objectives:** To evaluate the short- and medium-term effectiveness of self-administered acupressure taught by a short training course on reducing knee osteoarthritis (OA) pain in middle-aged and older adults.

**Hypothesis:** The self-administered acupressure group would have a greater pain relief compared to the knee health education (KHE) control in subjects with knee OA at week 12 (medium-term).

**Design and subjects:** In this fully-powered randomized controlled trial, 314 participants with knee OA recruited from the community will be randomized to self-administered acupressure or KHE group in a 1: 1 ratio.

**Study instrument:** Numerical rating scale (NRS) will be used to assess pain intensity in the knee.

**Interventions:** Participants in the self-administered acupressure will attend two 2-hour training sessions (1 week apart) to learn self-administered acupressure and practice twice daily for 12 weeks. Participants in the control group will receive knee health education of the same schedule and duration.

Main outcome measures: The primary outcome measure is the NRS score at 12 weeks. Other outcomes include Western Ontario and McMaster University Osteoarthritis Index, Short Form Six Dimensions, Timed Up & Go Test, Fast Gait Speed, pain medication usage. Compliance of self-administered acupressure will also be evaluated.

**Data Analysis:** Differences in the scale scores and test parameters will be examined using a linear mixed-effects model.

**Expected results:** Subjects in the self-administered acupressure group will have greater improvement in NRS and other related parameters compared to those in the KHE group at week 12.

## 1. Introduction

Knee osteoarthritis (OA) is a debilitating condition that commonly affects people aged over 50 years and 16% of people aged 45 or above were influenced (1). Acupuncture is well-evidenced for the effective treatment of knee OA pain (5); whereas acupressure, a non-invasive variant of acupuncture, is relatively less researched. Acupressure stimulates the same acupoints as acupuncture with the use of fingers, hands, or elbow based on traditional Chinese medicine (TCM) meridian theory. To facilitate a timely and more frequent intervention, acupressure can be self-administered by patients with knee OA after proper training, so that they may perform the acupressure after waking up in the morning or after a long walking in which the pain is usually more prominent.

We conducted a pilot RCT to test the feasibility and explore the effects of the self-administered acupressure on empirical acupoints to relieve knee OA pain using knee health education as a control from May to August 2017 (ClinicalTrials.gov identifier: NCT03155737). The subjects received either two lessons of self-administered acupressure training (n=17) or knee health education (n=18). They were phoned twice per week to remind their practice and answer queries during the 6-week study period. All subjects in the self-administered acupressure group had learned the self-acupressure technique and passed fidelity check after the course; the compliance was good (15 out of 17 subjects had attended all lessons). 83.3% of them had practiced the acupressure 5 days or more per week during the 6-week study period. The self-administered acupressure group showed a mean reduction of 2.27 points (SD=1.2) in NRS of pain scale and the total score appeared to be lower than the knee health education group ($d$=0.49), though they were not statistically significant due to the small sample size.

## 2. Aims and Hypotheses:

We hypothesize that the self-administered acupressure group would have a greater pain relief compared to the knee health education control in subjects with knee OA at week 12 (medium-term).

The specific aims are:

1. To examine the short- and medium-term effectiveness of self-administered acupressure taught by a short training course on relieving knee OA pain in middle-aged and older adults.
2. To evaluate the subjects' compliance with self-administered acupressure for knee OA.

To achieve these aims, we will perform an RCT on self-administered acupressure for alleviating knee OA pain delivered through a short training course using a knee health education course as a control.

**3. Subjects**

This RCT will recruit 314 participants from the community through advertisement in community centres, radio Health Programme, and newspapers as well as mass email at the University of Hong Kong and the Hong Kong Polytechnic University. Interested participants who provide written informed consent will be further examined for inclusion and exclusion criteria by a trained research assistant.

Inclusion criteria: 1. ethnic Chinese; 2. aged 50 years or above; 3. ability to comprehend Chinese; 4. fulfilling any 3 of the following criteria: i. morning stiffness ≤ 30 min; ii. crepitus on active joint motion; iii. bone tenderness; iv. bone enlargement; or v. no palpable joint warmth (This classification yielded 84% sensitivity and 89% specificity for OA knee diagnosis (14)); 5. having knee pain for at least 3 months (15); 6. Knee pain ≥3 on a Likert pain scale from 1–10; 7. having a smartphone (or a family member living together having a smartphone) that is compatible to WhatsApp (it is our experience in the pilot study that most of every subject had been used to using WhatsApp for social communication); and 8. willing to provide informed consent.

Exclusion criteria: 1. medical diagnoses or conditions that preclude individuals from active participation (e.g. bleeding disorders, alcohol or drug abuse); 2. knee pain related to other conditions (cancer, fracture, rheumatoid arthritis, rheumatism) as screened according to the red flags for further investigation or referral in the NICE 2014 Guidelines for Osteoarthritis of the knee (16); 3. score < 22 in Hong Kong Montreal Cognitive Assessment (HK-MoCA) indicating cognitive impairment that may prevent understanding of training instructions (17); 4. body mass index over 30, the obese II criteria for Asians (18) (it will be too difficult for the obese subjects to perform acupressure on the acupoints as physical pressure reaching the muscle is required); 5. presence of skin lesions or infections at the treatment sites; 6. ever had knee replacement surgery; 7. pregnancy or contemplating pregnancy, and 8. ever received acupressure or steroid injection for knee pain over the past 6 months.

Further investigation or referral, either at the screening or during the study, will be made when necessary. The CoA, a registered physiotherapist with a Master degree in Musculoskeletal Medicine and Rehabilitation and over 10 years clinical experience, will provide coaching in the screening and provide clinical opinions if necessary.

<u>Methods</u>

The proposed study is a **randomized controlled trial (Figure 1)**. Participants will be randomly assigned to receive 2 training sessions (2 hours each) of either

**self-administered acupressure** or **knee health education** in 1:1 ratio. Participants in the self-administered acupressure group will be told to perform the self-administered acupressure twice a day for 12 weeks, and participants in the knee health education group will follow the knee health instruction every day for 12 weeks. Reminders for practice will be sent to the participants via WhatsApp. Outcome measures will be assessed at baseline, week 4, 8, and 12. Ethical approval from the institute will be sought before commencement of the study. We will follow the CONSORT and STRICTA for the report of the trial and acupressure protocol.

Study design

Participants will attend two weekly 2-hour of self-administered acupressure training or knee health education according to the group allocation in a classroom at the School Nursing, the Hong Kong Polytechnic University. The participants will be trained to perform self-administered acupressure by an acupuncturist or receive knee health education by a registered nurse. To enhance interaction and ensure the quality of teaching, each class will be conducted in a small group of 4 to 6 subjects.

Treatment protocol

*Self-administered acupressure treatment group*

Participants in this group will receive two acupressure training sessions (2 hours each, 1-week apart). The self-administered acupressure treatment protocol was developed based on TCM meridian theory by the previous studies (10, 11) and modified by the PA who is an experienced acupuncturist. The acupoints are indicated for knee pain and have been commonly used (13). The protocol was tested for feasibility in our pilot study. We have modified the treatment protocol after the pilot study to further enhance the effectiveness and compliance of acupressure, including sending video on steps of locating acupoints and technique of acupressure to the participants via WhatsApp and incorporating a brief knee health education in the second session.

During the **1st session** (initial training), each participant will then receive a handout and an acupressure logbook. The handout includes a picture-illustrating acupressure step-by-step protocol. The acupressure logbook is for them to record their acupressure practice every day. The instructor will give a brief introduction about acupressure (20 minutes), followed by a group training on locating acupoints and manipulating acupressure technique (30 minutes). The treatment protocol consists of 4 steps (10, 11), namely warm-up, acupressure (on acupoints ST34, ST35, ST36, SP9, SP10, GB34, EX-LE2, and EX-LE4), rubbing the knee, and move the knee. The acupressure exercise takes about 16 minutes. The participants will then practice the acupressure (30 min), and then the instructor will inspect their performance of acupressure using a

competency checklist (30 minutes). The instructor will give feedback to their practice. Finally, the instructor will give a summary and answer queries (10 min). Additional training and practice time may be given to those who have not met the requirement in the checklist.

Participants will be asked to perform the acupressure exercise at home in the morning (within 1 hour after wake up) and night (within 1 hour after dinner) every day for 12 weeks. Participants will receive 2 follow-up phone calls from the instructor during the first week to remind their practice and answer their queries.

The **2nd session** (refreshing training) will be conducted 1-week after the first session ($2^{nd}$ week). The instructor will **revisit** the theory of acupressure and addressed issues raised by the subjects during their past week at-home practice (20 minutes). Participants' acupressure logbook will also be examined. The participants will be asked to have a **group practice** the self-acupressure (30 minutes), followed by a **brief knee health education** (30 minutes). Finally, the training will be concluded by a final practice and **fidelity check** by the instructor (30 minutes) and **Q&A** (10 minutes).

Fidelity of self-administered acupressure training by instructors

All the training materials will be reviewed and approved by the research team. A registered Chinese medicine practitioner with at least 5 years' clinical experience and at least a Master's degree will be recruited as an instructor to teach self-administered acupressure. The PA who is a senior acupuncturist, will go through the training materials with the instructor to ensure he/she understands the content and any pertinent details specific to the protocol. The instructor will run a demonstration course so that the PA and Co-A can assess whether the course content is being conveyed properly. PA will visit the first four classes and then randomly visit (at least one per month) to ensure the training content is adherence to the research protocol.

Fidelity of self-administered acupressure in participants

The capability of participants for self-administered acupressure will be inspected by the instructor with a **competency checklist** in each session. The practice of self-administered acupressure will be assessed in four domains: accuracy of locating acupoints, acupressure technique, strength, and frequency of acupressure at each acupoint. Advice and correction will be made when necessary. Video on steps of locating acupoints and instruction on the technique of acupressure will be sent to the participants via WhatsApp for their reference at home. A contact phone number will be available for participants to contact the research assistant if they have any queries regarding the acupressure. Participants' acupressure technique will be assessed again at week 4.

Monitoring of compliance at home (Intervention group)

Participants will receive an acupressure logbook to record the time and duration of their daily practice. Checklists for the correct performance of each step or skill will be available with the acupressure logbook to maximize the fidelity of self-administered acupressure. Telephone follow-up will be conducted by the instructor to remind them to practice and answer queries regarding the acupressure during the first week. Reminders will be made twice a week via WhatsApp by the research assistant during the remaining study period to remind subjects to practice self-acupressure.

*Knee health education control group*

Participants in this group will receive two sessions (2 hours each) of health education related to knee OA symptom management. The course content is developed from the course materials from the websites of Elderly Health Service, Department of Health, Hong Kong SAR (19) and reviewed by the CoA who is a physiotherapist.

During the 1st session, the instructor will invite the participants to share their knee problems and their experiences (30 minutes). The education will cover introduction to the causes, presentation and symptoms of knee OA (30 minutes). Prevention and self-care for the deterioration of knee OA will be introduced and explained to the participants (30 minutes). At the end of the session, all the subjects were encouraged to ask questions regarding the education content (15 minutes). The instructor will conclude the session with a summary and ask the participants to follow the prevention and self-care instruction taught in the class (15 minutes). A handout summarized the content of health talk and a logbook for recording daily pain management will be distributed to the participants.

During the 2nd session, the participants will be encouraged to raise questions regarding their practice of knee health instruction at home and difficulties came across during the past week (15 minutes). Followed by a revision of the content of the first session (30 minutes). The instructor will introduce available treatments and routine care for knee OA, including exercise, weight loss, medications, and diet regimen of Chinese medicine (30 minutes). The participants will have a 10-items multiple choices test to ensure they have understood the course content. The instructor will go through each item with explanation on the correct answer (30 minutes). The training session will be adjourned after all participants demonstrate an understanding of the instructions after a final Q&A section (15 minutes).

As a compensation, participants in knee health education group will receive the same self-administered acupressure training after completion of the 12-week assessment.

Monitoring of compliance at home (Control group)

Similar to the self-administered acupressure group, telephone follow-up will be conducted by the instructor to remind them to follow the instruction and answer queries regarding the education content during the first week; reminders will be made twice a week via WhatsApp by the research assistant during the remaining study period to remind subjects to follow the instruction. Participants will record whether they have followed the knee health instructions in a logbook.

Rationale of the control group

Since acupressure training involves much contact time between the instructor and participants, the improvement in participants' pain, if any, may be attributed to non-specific effects from the subject-practitioner interaction. Care as usual and waitlist control used in previous studies are not able to control for such non-specific effects and even lead to nocebo effects due to "not being treated". Therefore, in this proposed study, knee health education for the same length as in the self-administered acupressure group is adopted to control the contact hours between the instructor and participants. If our self-administered acupressure training is found to be more effective than education, then it can be examined in further studies to compare with other standard interventions or exercise programs.

Concomitant treatments

All the participants are allowed to keep their routine medical care for knee osteoarthritis provided by the public or private healthcare service in Hong Kong.

Methods of randomization and allocation concealment

Block randomization with a random block size of 4 to 6 will be used to randomize eligible participants equally into the two groups. A computer-generated list of numbers known only to an independent administrator will be used in the randomization process. The group allocation will be enclosed in sequentially numbered opaque sealed envelopes. After the participants have finished all baseline assessments and the blinded assessor has confirmed their eligibility, then the independent administrator will give the participants a sealed envelope. The participants will then open the envelope to reveal their group allocation. The research assistant who is responsible for phone-call follow-up will then look at their opened envelopes to log the participants' code and allocation.

Methods of blinding

The researchers who perform the assessment and analysis will be blinded to group allocation.

## 4. Outcome Assessment

Subjects will be assessed at baseline, week 4, 8, and 12 (**Table 1**).

- Primary Outcome Measure

-Pain severity numerical rating scale

The NRS indicates the pain intensity in the recent one week in the knee. NRS is a single 11-point numeric scale ranges from 0 (no pain) to 10 (greatest pain imaginable). The NRS is a valid and acceptable measure to detect changes in patients' improvement (20). NRS is chosen as primary outcome because our pilot suggested that the acupressure treatment protocol is more likely to improve global pain intensity. NRS has been used as the primary outcome in recent large scale RCTs of acupuncture and procedural interventions for knee OA.

- Secondary Outcome Measures

-Western Ontario and McMaster University Osteoarthritis Index (WOMAC)

The WOMAC covers pain, physical function, and stiffness related to knee osteoarthritis. It assesses pain and stiffness in specific activities as well as the level of physical function under different activities. WOMAC provides information complemented to NRS. The Chinese version of WOMAC has been validated (21).

-Short Form 6D (SF-6D)

The Short Form-6D is a preference-based measure of health derived from a selection of SF-36 items for economic evaluation. The SF-6D asks six multi-level dimensions: physical functioning, role limitations, social functioning, pain, mental health and vitality. The Chinese version of SF-6D was tested to be reliable and valid (22).

-Timed Up & Go Test (TUG)

The TUG test was developed and modified from a clinical measure of balance. Duration of a participant taken for completing the whole set of movements (stand up from armchair, walk for 3 miles, turn around, walk back and sit down) will be timed to reflect the knee function (23). Participants will be required to perform twice with 1-minute rest. The mean time will be calculated for analysis.

-Fast Gait Speed (FGS)

The fast gait speed (FGS) tests the time of crossing a marked 10-meter distance at a speed "as quickly as possible but safe" (24). Three consecutive trials will be conducted without walking aid and 1-minute rest will be given between trials. The time spent in each trial will be the averaged.

-Medication Use

Participants were permitted to continue using routine medications and pain medications, and maintain their usual physician visits throughout the study. We will

keep a written record the use of analgesics in a logbook throughout the study period.

Assessment of compliance

Participants' attendance at the training course will be recorded. To assess the compliance with home practice of self-administered acupressure, participants will be given an **acupressure logbook** to record their daily practice, including frequency and time spent on practicing self-administered acupressure during the 12-week study period. Similarly, participants in the control group will receive a **knee health logbook** to record their daily compliance to each instruction by yes/no questions. The same follow-up procedure will be conducted to assess their compliance.

Assessment of acceptability and feedbacks

The investigator-designed Evaluation Questionnaire was administered to both groups to evaluate the participants' feedback on the training course. The questionnaire includes a 10-point single item-scale (ranging from 1 to 10) to assess the participants' acceptability of self-administered acupressure or knee health education. Open-end questions will also be included to collect qualitative feedback regarding the best parts of the training and suggestions for any improvement.

Assessment of safety

i. Reasons for withdrawal

When a subject withdraws before completing the study, the reasons for withdrawal will be recorded.

ii. Adverse events

The instructor will ask, using open-ended questions, whether the participants experienced any adverse events during the assessment visit for immediate action and follow-up. The severity and relationship of the adverse event to the intervention will be investigated and documented.

Assessment of costs

Cost evaluation will be conducted to record the additional costs of implementing the self-acupressure training course for knee OA in the community centre setting from the perspectives of health service providers. Total costs will be summation of interventional costs, direct medical costs and direct non-medical costs.

Interventional costs will be included such as staff costs for organizing the course and running the course, telephone follow-up after the course, teaching materials, consumable materials, and rental cost of venue. To estimate the time cost of staff for organizing and preparing the course, the time spent on the program by each staff member (instructor, course assistant, and research assistant) will be recorded for

each course. The average number of minutes spent will be multiplied by the relevant salary rate per minute. The cost of teaching materials including the handouts, and other consumable will also be calculated. Besides, direct medical costs will account for the number of healthcare service visits during the trial period, whereas the direct non-medical costs will estimate transportation costs related to intervention and course attendance.

## 5. Data processing and analysis

*Sample size calculation*

Results from our pilot study (N=35) of self-administered acupressure for knee OA are used for sample size calculations. At week 6, the mean (SD) of NRS in the self-administered acupressure group and knee health education group was 2.83 (1.47) and 2.07(1.58), respectively. It equals to an effect size of 0.49. With a more conservative approach, we use 80% of the effect size, which is 0.40 for the sample size calculation. A sample size of 133 subjects per group can provide 90% power to reject the null hypothesis with a significance level of 0.05. Assuming an attrition rate of 15%, a total of 314 subjects is required for this study.

*Data management and analysis*

All data will be double entered. All statistical analysis will be performed in SPSS (23.0) for Windows. Subjects' sociodemographic, clinical characteristics and baseline data was examined for potential group difference and any identified baseline difference was controlled as covariates. The changes of the primary outcome (NRS score) and other secondary outcomes were compared using linear mixed effects model with group (self-administered acupressure and knee health education) by time point interaction (baseline to week 12). Acceptability and compliance will be presented using descriptive statistics. Clinical significance will be examined by the proportion of participants who have a reduction of at least 2 points from baseline in the NRS using Chi-square test (25). The intention-to-treat analysis will be conducted.

*Economic analyses*

Quality-adjusted life-years (QALYs) will be obtained from the SF-6D (22). Cost evaluation will be conducted to record the costs of implementation in the community setting from the perspectives of health service providers. Incremental costs and incremental effects will be compared between the self-acupressure and KHE groups. The incremental cost-effectiveness ratio (ICER) will be calculated by: Δ costs/Δ effects, where Δ costs represents the difference in the costs between self-acupressure and KHE groups, and Δ effects represents the difference in the QALYs (changes in SF-6D scores) between the two groups.

To test the robustness of the ICER and quantify the uncertainty in these ratios, bootstrapping involving 5,000 iterations will be performed and the results will be

plotted in a cost-effectiveness plane. A cost-effectiveness acceptability curve displaying the probability that the intervention is cost effective for a range of willingness-to-pay ceilings will also be estimated by using the bootstrapped ICERs in probabilistic sensitivity analysis. To confirm the robustness of the base-case findings, scenario analysis will be conducted by testing several assumptions made in the base-case scenario. Impacts of these assumptions on the ICER will be assessed.

## 6. Purpose and Potential

The findings of this project will be critical in determining the clinical effectiveness of offering a short acupressure training course to teach middle-aged and older to perform self-administered acupressure to relieve their knee pain. Knee OA is a prevalent condition leading to many negative health consequences, and it is expected to be more prevalent due to the aging population.

Self-administered acupressure is less time-consuming and at minimal cost. If found to be effective, we can recommend self-administered acupressure as an evidence-based strategy to healthcare providers for relieving knee OA pain in the community. Effective self-administered acupressure treatments could be made available to people knee OA in the community as an adjunct intervention to their current pain therapy and probably reducing their consumption of painkillers and prevent deterioration of their condition. The self-administered acupressure can be taught by Chinese medicine practitioners and other healthcare professionals such as nurses or physiotherapists. This cost-effective method will significantly relieve the burden on the public healthcare system. Healthcare providers, public health policy-makers, and individuals with knee OA will greatly benefit from the findings of this study.

## 7. Ethical approval

The protocol will be submitted to the Hong Kong Polytechnic University institutional review board and Institutional Review Board of the University of Hong Kong/ Hospital Authority Hong Kong West Cluster for approval. The study will be conducted according to the “Declaration of Helsinki”. Participation in the study is voluntary. An information sheet is provided and a written consent is required from the participant.

## 8. Potential hazards to participants

Since both self-administered acupressure and knee health education are non-invasive, the problem of adverse event will be minimal. The treatments used in the protocol have been tested in the pilot study and no participant had reported significant side effects. It is likely that the subjects experience slight discomfort when they are requested to apply pressure on the acupoints. Such a phenomenon will be explained

to the subjects in advance and the pressure will only be applied for a short period of time, which would obviously not produce any injury to the subjects. We will have phone calls and Whatsapp reminders (twice per week) and the subjects can raise questions if they have encountered any discomfort. The subjects can also call our Research Assistant at any time to have our assistance.

**Key References**

1. Jordan JM, Helmick CG, Renner JB, Luta G, Dragomir AD, Woodard J, et al. Prevalence of knee symptoms and radiographic and symptomatic knee osteoarthritis in African Americans and Caucasians: the Johnston County Osteoarthritis Project. J Rheumatol. 2007;34(1):172-80.
2. Peat G, McCarney R, Croft P. Knee pain and osteoarthritis in older adults: a review of community burden and current use of primary health care. Ann Rheum Dis. 2001;60(2):91-7.
3. McKenzie S, Torkington A. Osteoarthritis - management options in general practice. Aust Fam Physician. 2010;39(9):622-5.
4. Yang S, Dubé CE, Eaton CB, McAlindon TE, Lapane KL. Longitudinal use of complementary and alternative medicine among older adults with radiographic knee osteoarthritis. Clin Ther. 2013;35(11):1690-702.
5. Vickers AJ, Vertosick EA, Lewith G, MacPherson H, Foster NE, Sherman KJ, et al. Acupuncture for Chronic Pain: Update of an Individual Patient Data Meta-Analysis. J Pain. 2017;S1526-5900(17):30780 [Epub ahead of print].
6. Weerapong P, Hume PA, Kolt GS. The mechanisms of massage and effects on performance, muscle recovery and injury prevention. Sport Med. 2005;35(3):235-56.
7. Lee EJ, Frazier SK. The efficacy of acupressure for symptom management: a systematic review. J Pain Symptom Manage. 2011;42(4):589-603.
8. Chen YW, Wang HH. The effectiveness of acupressure on relieving pain: a systematic review. Pain Manag Nurs. 2014;15(2):539-50.
9. Song HJ, Seo HJ, Lee H, Son H, Choi SM, Lee S. Effect of self-acupressure for symptom management: a systematic review. Complement Ther Med. 2015;23(1):68-78.
10. Zhang Y, Shen CL, Peck K, Brismée JM, Doctolero S, Lo DF, et al. Training Self-Administered Acupressure Exercise among Postmenopausal Women with Osteoarthritic Knee Pain: A Feasibility Study and Lessons Learned. Evid Based Complement Alternat Med. 2012;2012:570431.
11. Sorour AS, Ayoub AS, Abd El, Aziz EM. Effectiveness of acupressure versus isometric exercise on pain, stiffness, and physical function in knee osteoarthritis female patients. J Adv Res. 2014;5(2):193-200.
12. Li LW, Harris RE, Tsodikov A, Struble L, Murphy SL. Self-Acupressure for Older Adults with Symptomatic Knee Osteoarthritis: A Randomized Controlled Trial. Arthritis Care Res. 2017:[Epub ahead of print].
13. Hou PW, Fu PK, Hsu HC, Hsieh CL. Traditional Chinese medicine in patients with

osteoarthritis of the knee. J Tradit Complement Med. 2015;5(4):182-96.

14. Altman R, Asch E, Bloch D, Bole G, Borenstein D, Brandt K, et al. Development of criteria for the classification and reporting of osteoarthritis: classification of osteoarthritis of the knee. Arthritis Rheum. 1986;29(8):1039-49.

15. MacPherson H. Pragmatic clinical trials. Complement Ther Med. 2004;12(2-3):136-40.

16. National Clinical Guideline Centre. Osteoarthritis: Care and Management in Adults. London: National Institute for Health and Care Excellence (UK); 2014.

17. Wong A, Xiong YY, Kwan PW, Chan AY, Lam WW, Wang K, et al. The validity, reliability and clinical utility of the Hong Kong Montreal Cognitive Assessment (HK-MoCA) in patients with cerebral small vessel disease. Dementia and Geriatric Cognitive Disorders. 2009;28(1):81-7.

18. Anuurad E, Shiwaku K, Nogi A, Kitajima K, Enkhmaa B, Shimono K, et al. The new BMI criteria for asians by the regional office for the western pacific region of WHO are suitable for screening of overweight to prevent metabolic syndrome in elder Japanese workers. J Occup Health. 2003;45(6):335-43.

19. Hong Kong Special Administrative Region Government. Osteoarthritis of Knee: Elderly Health Service, Department of Health, the Hong Kong Special Administrative Region Government; [Available from: http://www.elderly.gov.hk/english/common_health_problems/bones_and_joints/osteoarthritisknee.html.

20. Hawker GA, Mian S, Kendzerska T, French M. Measures of adult pain: Visual Analog Scale for Pain (VAS Pain), Numeric Rating Scale for Pain (NRS Pain), McGill Pain Questionnaire (MPQ), Short-Form McGill Pain Questionnaire (SF-MPQ), Chronic Pain Grade Scale (CPGS), Short Form-36 Bodily Pain Scale (SF-36 BPS), and Measure of Intermittent and Constant Osteoarthritis Pain (ICOAP). Arthritis Care Res (Hoboken). 2011;63(S11):S240-S52.

21. Symonds T, Hughes B, Liao S, Ang Q, Bellamy N. Validation of the Chinese Western Ontario and McMaster Universities Osteoarthritis Index in Patients From Mainland China With Osteoarthritis of the Knee. Arthritis Care Res (Hoboken). 2015;67(11):1553-60.

22. Lam CLK, Brazier J, McGhee SM. Valuation of the SF-6D Health States Is Feasible, Acceptable, Reliable, and Valid in a Chinese Population. Value Health. 2008;11(2):295-303.

23. Podsiadlo D, Richardson S. The timed “Up & Go”: a test of basic functional mobility for frail elderly persons. J Am Geriatr Soc. 1991;39:142-8.

24. Fransen M, Crosbie J, Edmonds J. Reliability of gait measurements in people with osteoarthritis of the knee. Phys Ther. 1997;77:944.

25. Farrar JT, Young JPJ, LaMoreaux L, Werth JL, Poole RM. Clinical importance of changes in chronic pain intensity measured on an 11-point numerical pain rating scale. Pain. 2001;94(2):149-58.

**Figure 1. Study Flowchart**

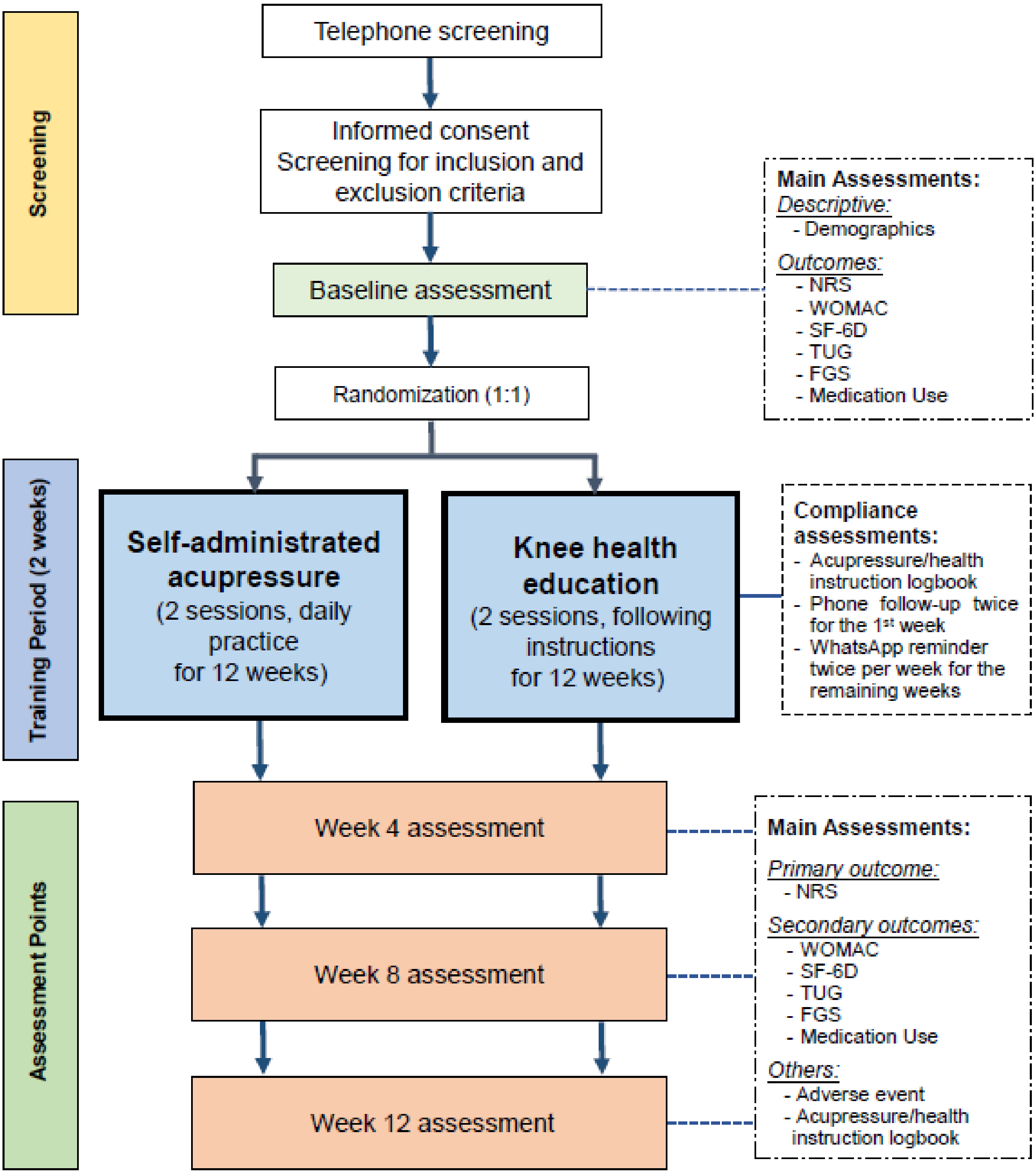


Abbreviation: NRS = Numerical Rating Scale; WOMAC = Western Ontario and McMaster University Osteoarthritis Index; SF-6D = Short Form Six Dimensions; TUG = Timed Up & Go Test; FGS = Fast Gait Speed.

**Table 1. Assessment Schedule**

| | Screening | Baseline | Training period (2 weeks) | | Assessment time points | | |
|---|---|---|---|---|---|---|---|
| **Week of study** | **-1** | **/** | **1** | **2** | **4** | **8** | **12** |
| | | | | | | | |
| Review inclusion/exclusion criteria | √ | √ | | | | | |
| Demographics | √ | | | | | | |
| Medical History | √ | | | | | | |
| Height & Weight | √ | | | | | | |
| Vital signs | √ | √ | √ | √ | √ | √ | √ |
| **Primary Outcome** | | | | | | | |
| Pain Severity Numerical Rating Scale | √ | √ | | | √ | √ | √ |
| **Secondary Outcomes** | | | | | | | |
| Western Ontario and McMaster University Osteoarthritis Index | | √ | | | √ | √ | √ |
| Short Form-Six Dimensions | | √ | | | √ | √ | √ |
| Timed Up & Go Test | | √ | | | √ | √ | √ |
| Fast Gait Speed | | √ | | | √ | √ | √ |
| Medication Use | | √ | | | √ | √ | √ |
| **Others** | | | | | | | |
| Acupressure/ health instruction logbook | | | √ | √ | √ | √ | √ |
| Adverse event monitoring | | | √ | √ | √ | √ | √ |
