# Supplementary material for: Self-Administered Acupressure for Probable Knee Osteoarthritis in Middle-Aged and Older Adults: A Randomized Clinical Trial
Source: JAMA Netw Open. 2024 Apr 19;7(4):e245830. doi: 10.1001/jamanetworkopen.2024.5830 (PMC11031685; doi:10.1001/jamanetworkopen.2024.5830)
Supplement: Supplement 2. — eFigure. Schedule of Self-Administered Acupressure Training Course and Knee Health Education Course eTable 1. Acupoints Used in the Treatment Protocol eTable 2. Self-Administered Acupressure Protocol eTable 3. Study Outcomes Across Study Time Points [file jamanetwopen-e245830-s002.pdf]

Supplemental Online Content

Yeung W, Chen S, Cheung DT, et al. Self-Administered Acupressure for Probable Knee Osteoarthritis in Middle-Aged and Older Adults. *JAMA Netw Open.* 2024;7(4):e245830. doi:10.1001/jamanetworkopen.2024.5830

eFigure. Schedule of Self-Administered Acupressure Training Course and Knee Health Education Course

eTable 1. Acupoints Used in the Treatment Protocol

eTable 2. Self-Administered Acupressure Protocol

eTable 3. Study Outcomes Across Study Time Points

This supplemental material has been provided by the authors to give readers additional information about their work.

© 2024 Yeung W et al. *JAMA Network Open.*

eFigure. Schedule of Self-Administered Acupressure Training Course and Knee Health Education Course

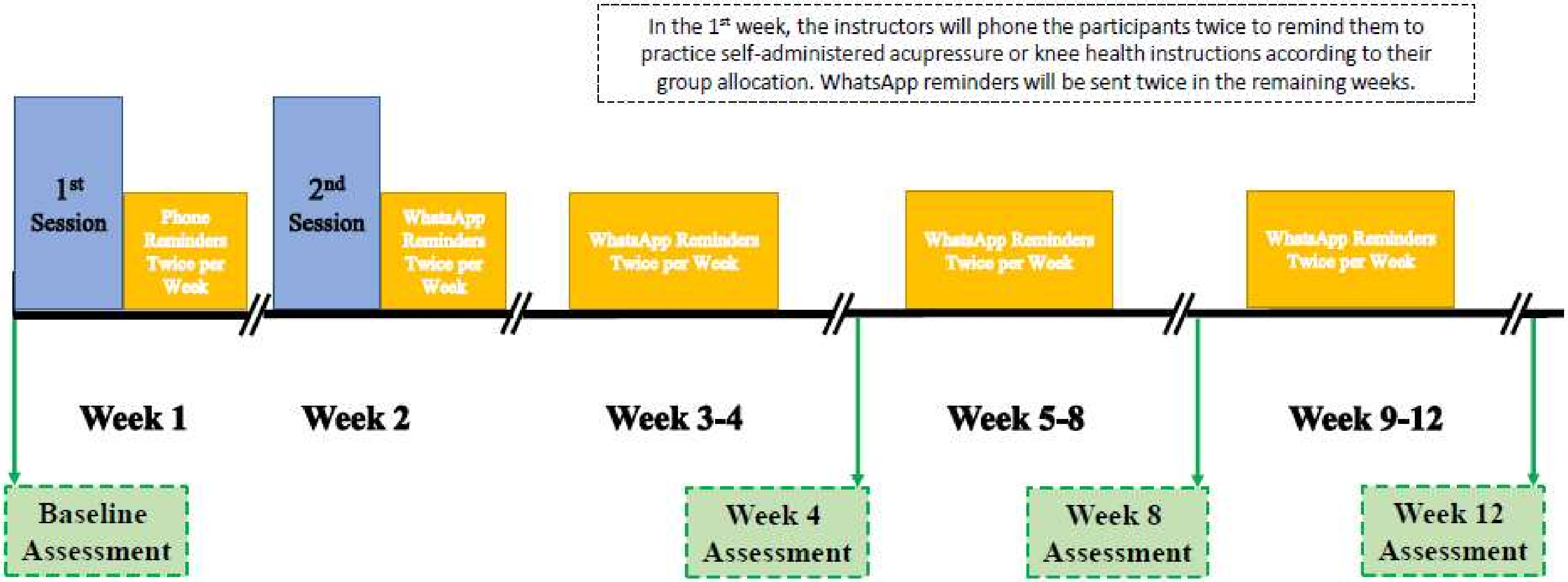

- **Content of self-administered acupressure training course**
  - **1st session:** Introduction (20 min); group training (30 min); practice (30 min); fidelity checking (30 min); Q&A (10 min).
  - **2nd session:** Revision (20 min); practice (30 min); brief knee health education (30 min); group practice and fidelity checking (30 min); Q&A (10 min).
- Participants will be told to practice the self-administered acupressure or knee health instructions **for 12 weeks.**

© 2024 Yeung W et al. *JAMA Network Open.*

eTable 1. Acupoints Used in the Treatment Protocol

Acupoints	Location	Function
Liangqiu (ST34, 梁丘)	With the knee flexed, on the anterior side of the thigh and on the line connecting the anterior superior iliac spine and the superiolateral corner of the patella, 2 *cun** above this corner	Used for paralysis and pain of the lower extremities, knee joint pain, gastritis, peptic ulcer disease.
Dubi (ST35, 犢鼻)	With the knee flexed, in the depression lateral and inferior to the patella and patellar ligament	Used for gonarthrosis, hemiplegia after stroke
Zusanli (ST36, 足三里)	On the anteriolateral side of the leg, 3 *cun* below *dubi*, one finger lateral to the anterior crest of the tibia	Used for hemiplegia after stroke, appendicitis, knee joint pain, gastritis, peptic ulcer disease, acute or chronic enteritis, dysentery
Yinlingquan (SP9, 陰陵泉)	On the medial side of the leg, in the depression posterior and inferior to the medial condyle of the tibia	Used for swelling and pain of the knee and leg, edema, urinary retention, enteritis, nephritis
Xuehai (SP10, 血海)	When the knee is flexed, on the medial side of the thigh, 2 *cun* proximal to the superinternal border of the patella, on the bulge of the medial portion of the quadriceps femoris of the thigh	Used for pruritus of skin, menoxenia, functional uterine bleeding, urticarial, knee pain
Yanglingquan (GB34, 陽陵泉)	On the lateral side of the leg, at the depression inferior and anterior to the head of the fibula	Used for paraplegia, sciatica, intercostal neuralgia, cholecystitis, biliary ascariasis, knee pain
He Ding (EX-LE2, 鶴頂)	In the depression of the midpoint of the superior patellar border	Used for inflammation and pain in the knee, weakness of the foot and leg
Xiyan (EX-LE5, 膝眼)	With the knee flexed, in the depression medial and inferior to the patella and patellar ligament	Used for swelling and pain of the knee joint, hemiplegia after stroke

* 1 *cun*: equals to width of subject's thumb

References

Zhang R. Introduction to acupuncture and moxibustion. Hackensack, N.J.: World Century Publishing Corporation; 2013.

Yang JD, Monti DA. Clinical Acupuncture and Ancient Chinese Medicine. New York: Oxford University Press; 2017.

© 2024 Yeung W et al. *JAMA Network Open.*

eTable 2. Self-Administered Acupressure Protocol

Components	Duration	Instruction
**Warm-up**	2 minutes	To use one hand to form a claw shape to squeeze then move along the front thigh (grasping quadriceps where meridians of *liver, spleen, stomach*, and *gallbladder* locate)
**Acupressure**	10 minutes	To use fingers/ thenar/ hypothenar to press the acupoints briskly then releasing with moderate pressure, repetitively for about 1 minute per acupoint (ST34, ST35, ST36, SP9, SP10, GB34, EX-LE2, and EX-LE4)<br>Details of the technique on each acupoint<br>• Thenar pressing for ST34 and SP10<br>• Fingers pressing with the emphasis of kneading was used for ST35 and EX-LE4<br>• Fingers pressing for ST 36 and SP 9<br>• Hypothenar pressing for EX-LE2
**Rubbing the knee**	2 minutes	To use one palm to cover the kneecap with the assistance of fingers to lift the kneecap lightly, and to move the palm gently in a small circle
**Move the knee**	2 minutes	To extend and rotate legs when participants sit on a chair with leg hanging by their hands holding their thighs

Self-administered acupressure will be suggested to perform on bilateral knees. If participants reported with only one knee is painful, the subjects may choose to perform on the painful knee only.

Reference: Zhang Y, Shen CL, Peck K, Brismée JM, Doctolero S, Lo DF, et al. Training Self-Administered Acupressure Exercise among Postmenopausal Women with Osteoarthritic Knee Pain: A Feasibility Study and Lessons Learned. Evid Based Complement Alternat Med. 2012;2012:570431.

© 2024 Yeung W et al. *JAMA Network Open.*

eTable 3. Study Outcomes Across Study Time Points

	Difference between groups in change between two time points (95% CI)	Effect size *d* [a]	P-value [b]
NRS Pain			
Week 4 to Week 8	-0.10 (-0.47, 0.28)	0.04	0.61
Week 4 to Week 12	0.04 (-0.38, 0.47)	-0.03	0.84
Week 8 to Week 12	0.12 (-0.27, 0.51)	-0.07	0.54
WOMAC – Pain			
Week 4 to Week 8	-0.17 (0.76, 0.41)	0.05	0.56
Week 4 to Week 12	0.17 (-0.49, 0.82)	-0.05	0.62
Week 8 to Week 12	0.38 (-0.16, 0.91)	-0.10	0.17
WOMAC – Stiffness			
Week 4 to Week 8	0.21 (-0.10, 0.53)	-0.13	0.19
Week 4 to Week 12	0.33 (-0.01, 0.67)	-0.20	0.06
Week 8 to Week 12	0.11 (-0.18, 0.40)	-0.07	0.45
WOMAC - Physical function			
Week 4 to Week 8	-0.82 (-2.66, 1.03)	0.07	0.39
Week 4 to Week 12	-0.28 (-2.31, 1.75)	0.01	0.79
Week 8 to Week 12	0.71 (-1.00, 2.43)	-0.06	0.41
TUG			
Week 4 to Week 8	-0.07 (-0.47, 0.34)	0.05	0.75
Week 4 to Week 12	0.17 (-0.23, 0.58)	-0.15	0.40
Week 8 to Week 12	0.35 (-0.01, 0.71)	-0.19	0.06
Gait Speed			
Week 4 to Week 8	-0.13 (-0.41, 0.15)	0.03	0.37
Week 4 to Week 12	-0.17 (-0.40, 0.06)	0.15	0.16
Week 8 to Week 12	-0.04 (-0.27, 0.19)	0.06	0.73
SF-6D			
Week 4 to Week 8	0.01 (-0.01, 0.03)	0.07	0.44
Week 4 to Week 12	0.02 (-0.004, 0.04)	0.16	0.10
Week 8 to Week 12	0.01 (-0.01, 0.03)	0.09	0.35

Abbreviation: SE, standard error; NRS, numerical rating scale; WOMAC, Western Ontario and McMaster University Osteoarthritis Index; TUG, Timed Up and Go Test; SF-6D, Short Form-Six Dimension.

[a] Effect size based on the mean change from previous assessment time point in the treatment group minus the mean change from previous assessment time point in the control group, divided by the pooled standard deviation. Effect size was considered as small, ES = 0.2, medium, ES = 0.5, and large, ES = 0.8 (Cohen, 1988). A positive sign denotes a better effect in the intervention group.

[b] P-value for interaction between groups and assessment time points in the linear mixed-effects model.

© 2024 Yeung W et al. *JAMA Network Open.*
