# Supplementary material for: Self-Administered Acupressure for Probable Knee Osteoarthritis in Middle-Aged and Older Adults: A Randomized Clinical Trial
Source: JAMA Netw Open. 2024 Apr 19;7(4):e245830. doi: 10.1001/jamanetworkopen.2024.5830 (PMC11031685; doi:10.1001/jamanetworkopen.2024.5830)
Supplement: Supplement 3. — Data Sharing Statement [file jamanetwopen-e245830-s003.pdf]

Data Sharing Statement

Yeung. Self-Administered Acupressure for Probable Knee Osteoarthritis in Middle-Aged and Older Adults. *JAMA Netw Open*. Published April 19, 2024.
doi:10.1001/jamanetworkopen.2024.5830

Data
Data available: Yes
Data types: Deidentified participant data
How to access data: jerry-wf.yeung@polyu.edu.hk
When available: With publication

Supporting Documents
Document types: None

Additional Information
Who can access the data: researchers whose proposed use of the data
Types of analyses: for secondary analyses
Mechanisms of data availability: after signed data access agreement
